# Temporal Dynamics of Soil Microbial Communities below the Seedbed under Two Contrasting Tillage Regimes

**DOI:** 10.3389/fmicb.2017.01127

**Published:** 2017-06-19

**Authors:** Florine Degrune, Nicolas Theodorakopoulos, Gilles Colinet, Marie-Pierre Hiel, Bernard Bodson, Bernard Taminiau, Georges Daube, Micheline Vandenbol, Martin Hartmann

**Affiliations:** ^1^Microbiology and Genomics, Department of AGROBIOCHEM, Gembloux Agro-Bio Tech, University of LiègeGembloux, Belgium; ^2^TERRA-AgricultureIsLife, Gembloux Agro-Bio Tech, University of LiègeGembloux, Belgium; ^3^Exchanges Ecosystems – Atmosphere, Department of BIOSE, Gembloux Agro-Bio Tech, University of LiègeGembloux, Belgium; ^4^Crop Sciences, Department of AGROBIOCHEM, Gembloux Agro-Bio Tech, University of LiègeGembloux, Belgium; ^5^Food Microbiology, University of LiègeLiège, Belgium; ^6^Forest Soils and Biogeochemistry, Research Institute for Forest, Snow and Landscape Research WSLBirmensdorf, Switzerland

**Keywords:** conventional tillage, reduced tillage, crop residue management, cropping season, microbial diversity, metabarcoding

## Abstract

Agricultural productivity relies on a wide range of ecosystem services provided by the soil biota. Plowing is a fundamental component of conventional farming, but long-term detrimental effects such as soil erosion and loss of soil organic matter have been recognized. Moving towards more sustainable management practices such as reduced tillage or crop residue retention can reduce these detrimental effects, but will also influence structure and function of the soil microbiota with direct consequences for the associated ecosystem services. Although there is increasing evidence that different tillage regimes alter the soil microbiome, we have a limited understanding of the temporal dynamics of these effects. Here, we used high-throughput sequencing of bacterial and fungal ribosomal markers to explore changes in soil microbial community structure under two contrasting tillage regimes (conventional and reduced tillage) either with or without crop residue retention. Soil samples were collected over the growing season of two crops (*Vicia faba* and *Triticum aestivum*) below the seedbed (15–20 cm). Tillage, crop and growing stage were significant determinants of microbial community structure, but the impact of tillage showed only moderate temporal dependency. Whereas the tillage effect on soil bacteria showed some temporal dependency and became less strong at later growing stages, the tillage effect on soil fungi was more consistent over time. Crop residue retention had only a minor influence on the community. Six years after the conversion from conventional to reduced tillage, soil moisture contents and nutrient levels were significantly lower under reduced than under conventional tillage. These changes in edaphic properties were related to specific shifts in microbial community structure. Notably, bacterial groups featuring copiotrophic lifestyles or potentially carrying the ability to degrade more recalcitrant compounds were favored under conventional tillage, whereas taxa featuring more oligotrophic lifestyles were more abundant under reduced tillage. Our study found that, under the specific edaphic and climatic context of central Belgium, different tillage regimes created different ecological niches that select for different microbial lifestyles with potential consequences for the ecosystem services provided to the plants and their environment.

## Introduction

It is well recognized that agricultural productivity strongly relies on a wide range of ecosystem services provided by the soil biota (Altieri, 1999). Although the delivery of ecosystem services are driven by complex interactions between the soil biota and abiotic parameters (Kibblewhite et al., 2008), most soil processes related to organic matter transformation and nutrient cycling are mediated by microorganisms (Nannipieri and Badalucco, 2003). Moreover, some specific symbiotic groups such as plant-growth promoting rhizobacteria and mycorrhizal fungi are well known to enhance crop productivity and plant health by stimulating plant growth and protecting plants against pathogens (Siddiqui et al., 2008; Berg, 2009). Microorganisms also contribute to soil aggregate formation and aeration, as well as carbon sequestration in agroecosystems (Six et al., 2006).

Plowing is one of the main components of conventional farming and has been used for centuries to control weeds, prepare the seedbed, temporary alleviate soil compaction, suppress soil-borne diseases, and improve nutrient mineralization and availability (Hobbs et al., 2008). Besides these short-term benefits, long-term detrimental effects such as soil erosion and loss of soil organic matter have been recognized (Six et al., 1999; Montgomery, 2007). Alternative soil management practices such as reduced or zero tillage, crop residue retention, mulching, crop rotation, and intercropping can significantly enhance both soil quality and crop productivity in agroecosystems (Scopel et al., 2012). In Belgian cropping systems, conventional tillage associated with crop residue exportation is currently the most commonly used tillage system applied by the farmers. Conventional tillage refers to soil inversion down to approximately 25–30 cm using a moldboard plow. Nowadays in Belgium, there is a growing interest to use alternative tillage systems that minimize soil disturbance. More specifically, the transition from conventional to alternative conservation tillage seems to be mostly driven by economy (Lahmar, 2010). In Belgium, however, there is still little scientific evidence that alternative tillage system indeed improves soil quality and crop productivity. The local climatic and pedological context are major factors driving the influence of the tillage regime on soil physical, chemical and biological properties. In our study, we compared conventional plowing system (CT) with reduced tillage (RT), an intermediate soil disturbance tillage system where only the top 10 cm of soil is disturbed in order to improve the conditions for seed germination.

Moving towards more sustainable agricultural management and more specifically towards reduced tillage with crop residue retention is not without consequences for the soil microbiota in terms of structure (α- and β-diversity) and functions. Several studies have reported effects of soil tillage and/or crop residue management on soil microbial community structures (Quadros et al., 2012; Navarro-Noya et al., 2013; Carbonetto et al., 2014; Sengupta and Dick, 2015; Degrune et al., 2016; Jiménez-Bueno et al., 2016). For example, the diversity of arbuscular mycorrhizal fungi (AMF), a group of fungi supporting the host plant with enhanced nutrient acquisition and increased resistance against drought and root pathogens (Van Der Heijden et al., 1998), has shown to be increased under reduced tillage (Säle et al., 2015). Other studies have shown that enzymatic activities related to soil organic C, N, P, and S cycling increased when applying principles of conservation agriculture such as zero-tillage and/or crop residue retention (Panettieri et al., 2014; Murphy et al., 2016). Residue retention also appeared to increase soil microbial biomass (Salinas-Garcia et al., 2001).

In general, the soil microbiota is affected by various abiotic factors such as pH (Lauber et al., 2009), soil moisture (Brockett et al., 2012), oxygen availability (Lüdemann et al., 2000), quality of organic substrates (Bending et al., 2002), nutrient inputs such as nitrogen and phosphorus (Leff et al., 2015), soil texture (Girvan et al., 2003; Chau et al., 2011) and temperature (Frey et al., 2008), as well as biotic factors such as plant communities (Kowalchuk et al., 2002) and the occurrence of other soil organisms such as earthworms (Héry et al., 2007). It is well established that many of these parameters are likely to change with tillage regime and crop residue management as reported by a large body of literature (e.g., Six et al., 1999; Lipiec et al., 2006; D’Haene et al., 2008; Murphy et al., 2016), which in return may influence soil microbial communities and the ecosystem services they provide.

In addition to the tillage regime and crop residue management, growing season of the crop is a major driver of microbial community structure in agricultural systems (Houlden et al., 2008; Lauber et al., 2013). Root system development over the growing season and associated changes in rhizodeposition may alter the spatial distribution and quality of organic materials (Philippot et al., 2013), influencing the dynamics of the microbial community over time. Although previous studies have investigated the growing season effect on microbial community structure, only few have looked at the dynamics under different soil treatments over the course of a growing season (Spedding et al., 2004; Zhang et al., 2012; Shi et al., 2013). These previous studies did not harness the information of the high-throughput sequencing technologies in order to assess such effects at a higher coverage and taxonomic resolution. Since individual members of the soil microbiota can have both beneficial and detrimental effects on crop growth and productivity, a detailed assessment of their specific response is of primary interest.

In the presented study, two different hypotheses have been tested: (1) below the seedbed, different tillage regimes alter the structure (α- and β-diversity) of microbial communities and lead to different microbial life strategies by changing soil physical and chemical properties, and (2) below the seedbed, tillage-related effects on soil microbial community structure vary across the growing season and differences in community structure between conventional and reduced tillage get smaller towards the end of the growing season. To test these hypotheses, we employed a 454 pyrosequencing approach of bacterial and fungal ribosomal markers to examine the response of soil microbial community structure to 6 years of continuous reduced and conventional tillage combined with residue retention or removal over the course of the growing season of two crops, i.e., *Vicia faba* (faba bean) and *Triticum aestivum* (wheat), in an experimental field located in central Belgium and characterized by a loess-derived soil. Understanding the microbial taxon-level response over the growing season in soils subjected to different management practices has the potential to optimize current agricultural practices in order to promote beneficial microorganisms and, thus, improve the sustainability of agriculture.

## Materials and Methods

### Site Description

The SOLRESIDUS long-term experiment, located on the experimental farm of Gembloux Agro-Bio Tech (University of Liège, Belgium, at 50°33′45.92″N and 4°42′48.97″E), is characterized by an oceanic temperate climate and a Cutanic Luvisol. The soil texture is silt loam and largely dominated by silt (70–80%), clay (18–22%), and sand (5–10%). The monthly average temperature is highest in July, at 18.4°C, and lowest in January, at 3.3°C. The monthly average rainfall is highest in December, at 81 mm, and lowest in April, at 51.3 mm (data from the Belgian Royal Meteorological Institute).

### Soil Treatments and Experimental Design

The experimental design consisted of a Latin square arrangement with four replicates of four soil treatments and has previously been described in detail (Degrune et al., 2016). Briefly, each soil treatment consisted of a combination of different soil practices: a tillage regime (conventional or reduced tillage) combined with a crop residue management practice (residue retention or removal). The combinations were as follows: conventional tillage with residue removal (CT/-R, the agricultural practice most commonly used in Belgium), conventional tillage with residue retention (CT/+R), reduced tillage with residue removal (RT/-R), and reduced tillage with residue retention (RT/+R). Conventionally tilled plots were plowed to a depth of 25 cm, while in the plots under reduced tillage only the top 10 cm of the soil was mixed (shallow tillage). The quantity of faba bean residue left on the field in 2013 was 6.4 t/ha under R+ and 3.1 t/ha under R-. For wheat, the quantity of crop residue left on the field was 9.4 t/ha under R+ and 4.7 t/ha under R-. Crops were rotated on the studied field and crop history is as follows: *Brassica napus* (2009), *T. aestivum* (2010, 2011, and 2012), *V. faba* (2013), and *T. aestivum* (2014).

### Soil Sampling and Soil Chemical Analysis

Soil samples were collected from each of the 16 plots in 2013 (*V. faba*) and 2014 (*T. aestivum*) at different growing stages including the seedling (early), leaf development (intermediate) and flowering stages (late) for *V. faba*, as well as tillering (early) and grain filling (late) stages for *T. aestivum*. Each soil sample corresponded to a composite of six randomly selected soil cores of 5 cm length and 2 cm diameter each and collected at 15–20 cm, i.e., below the seedbed. The effect of tillage on the topsoil microbiota during the late stage of winter wheat cultivation was explored in a previous study (Degrune et al., 2016). This study focused on the soil strata below the seedbed, since the nature of disturbance between CT and RT is most pronounced in this area. Below the seedbed, large differences between CT and RT were expected since the soil under RT was undisturbed for the last 6 years. The detail of field operations is provided in **Table 1**.

**Table 1:** Field operations performed on the SOLRESIDUS experiment in 2012 and 2013.

2012			2013			2014		
		
Date	Operation field	Plot	Date	Operation field	Plot	Date	Operation field	Plot
29/08	Shallow tillage	All	18/03	Weeding	All	11/03	Nitrogen fertilization	All
06/09	Cover crop sowing (mustard)	All	05/04	Sowing faba bean	All	26/03	Soil sampling	All
13/12	Plowing	CT	08/04	Meadow-emergence weeding	All	01/04	Weeding	All
			15/04	Soil sampling	All	15/04	Nitrogen fertilization	All
			24/05	Soil sampling	All	15/04	Growth regulator	All
			27/06	Soil sampling	All	25/04	Weeding	All
			08/07	Chemical pest control	All	27/04	Fungicide	All
			28/08	Weeding	All	12/05	Nitrogen fertilization	All
			04/09	Faba bean harvest	All	16/05	Weeding	All
			25/11	Plowing	CT	26/05	Soil sampling	All
			25/11	Shallow tillage	All	06/06	Fungicide	All
			25/11	Sowing winter wheat	All	04/09	Winter wheat harvest	All


Soil physical and chemical properties of each sample were determined as outlined in the following. Water content was measured by drying soil samples at 105°C during 48 h. Soil pH was measured in 1 M KCl (2:5 w:v) after 2 h of equilibration. Water-extractable elements were quantified by flame absorption (Ca, Mg), flame emission (P, Na), or colorimetry (P) after extraction of 20 g of 8-mm-sieved fresh soil in 100 ml H_2_O for 1 h at room temperature and filtration on 602 H 1/2. Hot water carbon was quantified as described by Ghani et al. (2003). Nitrates (NO_3_^-^) and ammonium (NH_4_^+^) were determined in 2 M KCl of soil extracts by flow injection analysis, using QuickChem^®^ (Method 12-107-06-3-B, Lachat instruments 5600 lindbergh drive Loveland, CO 80539 United States).

### Pyrosequencing of 16S and 28S rRNA Genes

DNA extraction and pyrosequencing of bacterial and fungal ribosomal markers were fully described by Degrune et al. (2016). Briefly, the V1-V3 region of the 16S rRNA gene (approximately 500 bp) and the D1–D2 region of the 28S rRNA gene (approximately 700 bp) were unidirectionally sequenced using the GS junior-FLX Titanium technology (Roche 454 Life Sciences, Brandford, CT, United States). Sequence data were processed according to Hartmann et al. (2014) including procedures to reduce the influence of sequencing errors (Quince et al., 2009), PCR substitution errors (Quince et al., 2009), and chimeras (Edgar et al., 2011) as implemented in mothur (Schloss, 2009), as well as target verification and extraction (Hartmann et al., 2010; Nilsson et al., 2010). Denoised sequences were clustered into operational taxonomic units (OTUs) using CROP (Hao et al., 2011) at 97% sequence identity. CROP center sequences were queried against SILVA (16S rRNA) and RDP (28S rRNA) (Maidak et al., 1996; Quast et al., 2012) using the naive Bayesian classifier (Wang et al., 2007) implemented in mothur and a minimum bootstrap support of 60%. Singletons, i.e., OTUs that contain only one sequence were removed prior to statistical analyses.

#### Statistics and Data Visualization

All statistical analyses were performed using Primer6+ (Clarke and Gorley, 2006) and the R software (R Development Core Team, 2011). Adjustments for multiple testing were performed using the false discovery rate correction according to Storey (2002) performed with qvality (Käll et al., 2009) unless indicated otherwise. We tested the effect of the following factors: *tillage regime* with conventional (CT) and reduced (RT) tillage, *crop residue management* with residue retention (R+) and removal (R-), *crop* with *V. faba* and *T. aestivum*, and the *growing season* of *V. faba*: seedling (early stage), leaf development (intermediate stage), flowering (late stage), and *T. aestivum*: tillering (early stage) and grain filling (late stage). Since the growing stages differed for each crop, this factor was nested in crop. Differences in β-diversity were examined using the Bray–Curtis similarity calculated from normalized and square-root transformed OTU abundances. The significance of the experimental factors was tested using multivariate permutational analysis of variance [PERMANOVA, Anderson (2001)] as implemented in Primer6+ with 99,999 permutations. The heterogeneity of variance between groups was tested using permutational analysis of dispersion [PERMDISP, Anderson (2001)] as implemented in Primer6+ with 99,999 permutations. The major variance components of bacterial and fungal β-diversity were visualized using principal coordinate analyses [PCO, Gower (1966)]. Estimates of α-diversity, i.e., observed richness Sobs and Smith-Wilson evenness E (Smith and Wilson, 1996), were based on evenly rarefied OTU abundance matrices using an iterative subsampling procedure with 1000 iterations as implemented in mothur. The significance of the experimental factors on α-diversity and soil physical and chemical parameters were examined using univariate PERMANOVA based on Euclidean distances calculated from z-transformed data as implemented in Primer6+ with 99,999 permutations. The relationship between the soil properties and microbial community structure was assessed using the distance-based linear modeling [DistLM, McArdle and Anderson (2001)] procedure implemented in Primer6+ with 99,999 permutations.

The response of individual taxa at high (phylum) and low (OTUs) resolution was evaluated using PERMANOVA as implemented in the *adonis* function of the R package vegan (Oksanen et al., 2007). In order to visualize positive or negative responses of the individual taxa to one of the tillage regimes, the relative abundances were z-transformed and then averaged by tillage. The same analysis was performed on the individual soil physico-chemical parameters. Taxonomic networks were used to visualize the OTU distribution across the taxonomic hierarchy (Hartmann et al., 2015; Frey et al., 2016). The response of the significant OTUs to tillage was represented by values derived from z-transformed data independent from the growing season (i.e., centered by stage), and ranged from -1 to 1. The network was generated in Cytoscape 3.3.0 (Shannon et al., 2003) using the *Allegro Fruchterman-Reingold algorithm* (Fruchterman and Reingold, 1991). The network is characterized by nodes (=OTUs) and edges (=taxonomic path from phylum to OTU level), whereas OTUs are placed at the level of the lowest possible taxonomic assignment. The response of individual OTUs to tillage was mapped onto the taxonomic network.

## Results

### Effect of Soil Management and Growing Season on β- and α-Diversity

**FIGURE 1:**
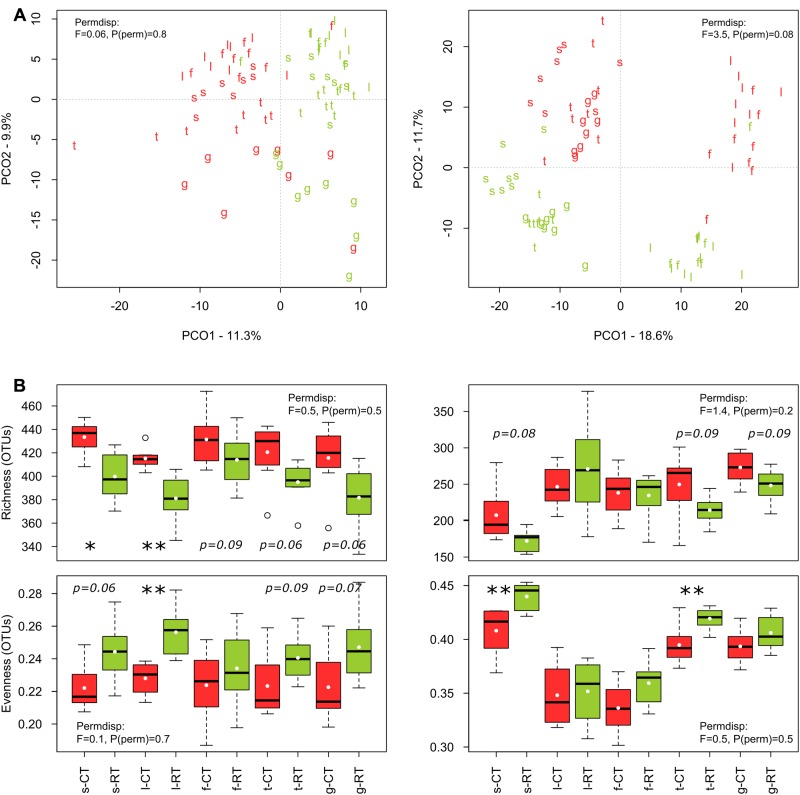
Effects of tillage regime and growing season on bacterial and fungal β- **(A)** and α-diversity **(B)**. The principal coordinate analyses (PCO) ordination axes PCO1 and PCO2 represent 11 and 10% of the bacterial community variation, respectively, and 19 and 12% of the fungal community variation, respectively. Tillage regime is represented by color code with CT = red and RT = green and growing season is represented by letters with *s* = seedling, *l* = leaf development, *f* = flowering, *t* = tillering, *g* = grain filling. *s*, *l*, and *f* correspond to early, intermediate and late stage of *V. faba*, and *t* and *g* correspond to early and late stage of *T. aestivum*. Test statistics for assessing the heterogeneity of variance for tillage effect as assessed by PERMDISP are provied in the plots. The significant changes are labeled by asterisks: ^∗^*p* < 0.05, ^∗∗^*p* < 0.01, ^∗∗∗^*p* < 0.001).

The sequencing runs yielded a total of 935,850 bacterial and 951,972 fungal raw reads, respectively. After quality filtering, a total of 393,004 (4,913 ± 1,887 per sample) bacterial 16S_V 2-V 3_ and 456,709 (5,709 ± 1,312 per sample) fungal 28S_D1_ high-quality sequences were obtained for the 80 soil samples, yielding a total of 1710 bacterial and 1567 fungal OTUs. Tillage regime (explaining 7–10% of the variance), crop (7–9%), and growing season (10–19%) emerged as important factors driving microbial β-diversity (**Table 2A**). Management of the crop residues showed no (bacteria) or only small (fungi) influence on β-diversity (**Table 2A**). These shifts in bacterial and fungal β-diversity due to tillage, crop and growing season became evident in the PCO plots, with communities clustering by tillage regime on the first (bacteria) or second (fungi) axis (**Figure 1A**). Compositional shifts due to crop and growing season became evident on the corresponding other main component. For bacteria, the compositional shift between *V. faba* and *T. aestivum* was evident on the second axis with the seedling (*s*), leaf development (*l*), and flowering (*f*) stages of *V. faba* separated from the growing stages of *T. aestivum*, i.e., tillering (*t*) and grain filling (*g*). In addition, a shift in the composition can be noticed among the growing stages of each crop. The fungal community showed a similar pattern; notably, however, the seedling (*s*) stage of *V. faba* clustered together with the growing stages of *T. aestivum* (*t* and *g*) likely caused by the preceding wheat cultivations.

**Table 2:** Effects of tillage regime, crop residue management, crop, and growing season on bacterial and fungal β-diversity.

	Bacteria			Fungi		
		
(A) *Main test*	*F*	*P*(perm)	*R*^2^	*F*	*P*(perm)	*R*^2^
Tillage	**6.9**	**^∗∗∗^**	**7**	**9.7**	^∗∗^	**10**
Residue	0.9	0.6	1	1.6	0.2	1
Crop	**6.6**	**^∗∗∗^**	**7**	1.4	0.2	9
Season	**3.2**	**^∗∗∗^**	**10**	**7.5**	**^∗∗∗^**	**19**
Tillage^∗^season	1.1	0.2	3	1.1	0.1	3

**(B)** ***Pairwise test***	***t***	***P*_adjust_**	**Avg sim**	***t***	***P*_adjust_**	**Avg sim**

s-CT, s-RT	**1.6**	^∗∗∗^	**69.9**	**1.8**	^∗∗∗^	**59.9**
l-CT, l-RT	**1.6**	^∗∗∗^	**67.0**	**2.1**	^∗∗∗^	**51.7**
f-CT, f-RT	**1.3**	^∗^	**73.3**	**1.5**	^∗∗^	**58.2**
t-CT, t-RT	**1.9**	^∗∗∗^	**71.3**	**1.9**	^∗∗∗^	**56.4**
g-CT, g-RT	1.1	0.1	69.3	**1.7**	^∗∗∗^	**58.7**


***(A)** Effects of main factors and their interactions as assessed by multivariate permutational analysis of variance (PERMANOVA). Main factors represent tillage (CT, RT), residue management (R+, R-), crop (*Vicia faba* and *Triticum aestivum*), and growing season (*s*, seedling; *l*, leaf development; *f*, flowering; *t*, tillering; *g*, grain filling). s, l, and f correspond to early, intermediate and late stage of *V. faba*, and t and g correspond to early and late stage of *T. aestivum*. Values represent the pseudo-*F* ratio (*F*), the permutation-based level of significance [*P*(perm)] and the proportion of variance explained by each factor (*R*^2^). **(B)** Pairwise comparisons between tillage regimes over the season of the two crops. Values represent the univariate *t*-statistic (*t*), the permutation-based level of significance adjusted for multiple comparisons using false discovery rate correction according to Benjamini–Hochberg (Benjamini and Hochberg, 1995) (*P*_*adjust*_), and the average between-group Bray–Curtis similarity (avg sim). Significant results are labeled in bold (^∗^*p* ≤ 0.05, ^∗∗^*p* ≤ 0.01, ^∗∗∗^*p* ≤ 0.001).*

Around 3% of the variance in bacterial and fungal β-diversity was explained by an interaction between tillage and growing season (**Table 2A**). This interaction became evident when examining the pairwise tests (**Table 2B**). For faba bean, the bacterial and fungal communities showed the highest dissimilarity (lowest similarity value) between CT and RT at leaf development (*l*), and the communities became again more similar at flowering stage (*f*). For wheat, the bacterial communities were different between CT and RT at tillering (*t*) stage but not at grain filling stage (*g*), whereas the fungal communities were distinct between CT and RT at both growing stages.

Bacterial α-diversity was mainly influenced by tillage regime, while fungal α-diversity was mostly influenced by the growing season (**Table 3**). In contrast with bacteria, fungal α-diversity was substantially influenced by the type of crop (**Table 3**). For bacteria, CT was more rich and less even than RT, while for fungi, richness remained similar between CT and RT, and CT was less even (**Figure 1B**).

**Table 3:** Effects of tillage regime, crop residue management, crop, and growing season on bacterial and fungal α-diversity.

	Bacteria		Fungi	
		
Main test	Richness (sobs)	Evenness (sw)	Richness (sobs)	Evenness (sw)
				
	*F*(*P*)	*F*(*P*)	*F*(*P*)	*F*(*P*)
Tillage	**36.3 (^∗∗∗^)**	**26.1 (^∗∗∗^)**	3.6 (0.06)	**16.5 (^∗∗∗^)**
Residue	0.08 (0.8)	0.8 (0.4)	0.5 (0.5)	2.2 (0.1)
Crop	3.6 (0.06)	0.1 (0.7)	**5.1 (^∗^)**	**38.0 (^∗∗∗^)**
Season	**4.3 (^∗∗^)**	1.6 (0.2)	**12.5 (^∗∗∗^)**	**45.8 (^∗∗∗^)**
Tillage^∗^season	0.6 (0.6)	0.8 (0.5)	2.0 (0.1)	1.5 (0.2)


*Effects of main factors and their interactions as assessed by univariate permutational analysis of variance (PERMANOVA). Main factors represent tillage (CT, RT), residue management (R+, R-), crop (*V. faba* and *T. aestivum*), and growing season (*s*, seedling; *l*, leaf development; *f*, flowering; *t*, tillering; *g*, grain filling). Seedling, leaf development and flowering correspond to early, intermediate and late stage of *V. faba*, and tillering and grain filling correspond to early and late stage of *T. aestivum*. Values represent the pseudo-*F* ratio (*F*) and the permutation-based level of significance (*P*). The significant results are provided in bold (^∗^*p* ≤ 0.05, ^∗∗^*p* ≤ 0.01, ^∗∗∗^*p* ≤ 0.001).*

As differences in α- and β-diversity between CT and RT can arise from differences in similarity, differences in dispersion or both, a separate test of dispersion using PERMDISP was used to detect the nature of such differences. Results reported no differences in dispersion, i.e., homogeneity of variance, suggesting that differences in α- and β-diversity were largely driven by dissimilarity rather than dispersion (see PERMDISP on **Figure 1**).

### Relationship between Soil Chemical Properties and Microbial β-Diversity

**FIGURE 2:**
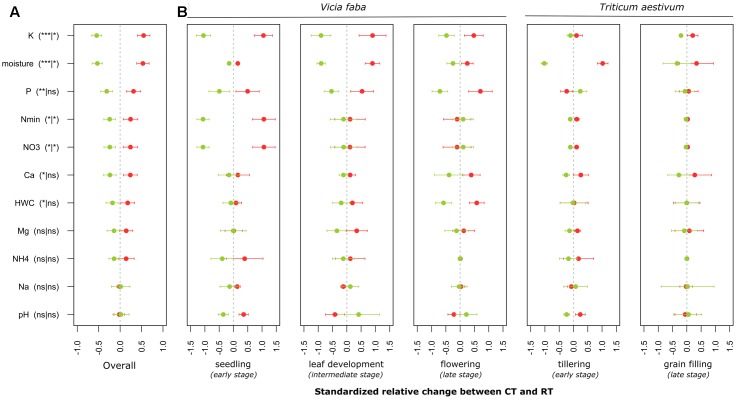
Standardized relative changes in physical and chemical soil properties combined over all growing stages **(A)** as well as for each individual stage **(B)** between CT (red) and RT (green). Data were z-transformed, representing values greater or smaller than the average across all samples. The significance of the PERMANOVA test is indicated in brackets: the first argument represents the significance of tillage effect and the second represents the significance of the interaction between tillage and growing stage. K, potassium; P, phosphorus; Nmin, mineral nitrogen; ${NO}_{3}^{-}$, nitrate; Ca, calcium; HWC, hot water carbon; Mg, magnesium; NH_4_^+^, ammonium; Na, sodium. CT, conventional tillage; RT, reduced tillage; ^∗∗∗^*q* < 0.001, ^∗∗^*q* < 0.01, ^∗^*q* < 0.05; ns, not significant.

Our findings identified crop as the main driver of the soil chemical properties (*F* = 39, *p* = 0.00001), growing season as the second (*F* = 14, *p* = 0.00001) and tillage regime as the third ranking factor (*F* = 6, *p* = 0.00001), while no crop residue effect (*F* = 0.9, *p* = 0.5) was observed. A low but significant interaction effect between growing season and tillage regime was noticed (*F* = 1.6, *p* = 0.03). Based on the main test provided for each soil parameter (**Table 4A**), we identified the levels of P, K, Ca, NO_3_^-^, Nmin and soil moisture to change with tillage regime. CT consistently showed higher levels of these properties when compared to RT (**Figure 2A**). Several parameters also revealed an interaction effect between the tillage and the growing season (**Table 4A** and **Figure 2B**), indicating significant variability in the tillage effect across the growing season.

**Table 4:** Effects of tillage regime, crop residue management, and growing season on soil physical and chemical soil properties.

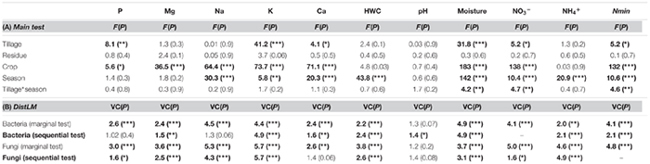

***(A)** Effects of main factors and their interactions as assessed by univariate permutational analysis of variance (PERMANOVA). Main factors represent tillage (CT, RT), residue management (R+, R-), crop (*V. faba* and *T. aestivum*), and growing season (*s*, seedling; *l*, leaf development; *f*, flowering; *t*, tillering; *g*, grain filling). s, l, and f correspond to early, intermediate and late stage of *V. faba*, and *t* and *g* correspond to early and late stage of *T. aestivum*. Values represent the pseudo-*F* ratio (*F*) and the permutation-based level of significance (*P*). **(B)** Distance based-linear analysis (DISTLM) between microbial β-diversity and soil parameters. Values represent the variance component (VC) and the permutation-based level of significance (*P*). The significant results are provided in bold (^∗^*p* < 0.05, ^∗∗^*p* < 0.01, ^∗∗∗^*p* < 0.001).*

The relationship between microbial community structure and soil chemistry was tested for each property separately (**Table 4B**, marginal test) as well as by fitting all predictors into the most parsimonious model (**Table 4B**, sequential test). The best model for bacteria revealed the combination of soil moisture, K, Nmin, NH_4_^+^, HWC, Mg, and pH as the best set of predictors (in decreasing order of importance) for explaining variations in community structure. For fungi, the model revealed K, soil moisture, Nmin, NO_3_^-^, and P as the best set of predictors (**Table 4B**).

### Individual Response of Taxa to Tillage Regime

**FIGURE 3:**
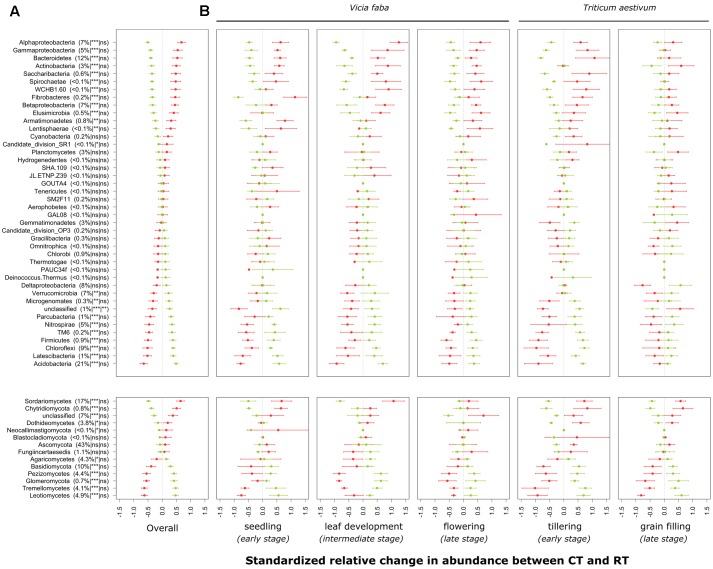
Standardized relative changes in abundance of higher-order taxonomic groups between CT (red) and RT (green) across all growing stages **(A)** and separately for each individual growing stage **(B)**. Data were z-transformed, representing values greater or smaller than the average across all samples. The relative abundance as well as the significance of the PERMANOVA test is indicated in brackets: the first argument represents the relative abundance, the second is the significance of tillage effect, and the third represents the significance of the interaction between tillage and growing stage. CT, conventional tillage; RT, reduced tillage; ^∗∗∗^*q* < 0.001, ^∗∗^*q* < 0.01, ^∗^*q* < 0.05; ns, not significant.

The individual relative change in abundance of higher-order taxonomic groups (phylum and major classes of Proteobacteria, Ascomycota, and Basidiomycota) to the tillage regime is shown in **Figure 3A**. Major groups of bacteria including Proteobacteria (α-, γ-, and β-Proteobacteria), Bacteroidetes and Actinobacteria increased in relative abundance under CT, whereas Acidobacteria, Chloroflexi, Nitrospirae, Verrucomicrobia, and δ-Proteobacteria increased in relative abundance under RT. In addition to these major groups, some bacterial candidate phyla including TM6 (recently called Dependentiae), Parcubacteria, Latescibacteria, and Microgenomates increased in relative abundance under RT, whereas Saccharibacteria increased under CT. In the same way, major groups of fungi including Sordariomycetes, Dothideomycetes and Chytridiomycota increased in relative abundance under CT, whereas the relative abundance of Agaricomycetes, Basidiomycota, Pezizomycetes, Glomeromycota, Tremellomycetes, and Leotiomycetes increased under RT. Tillage effects on higher-order taxonomic groups of bacteria and fungi showed a certain degree of variability over the growing season, although none of the bacterial phyla or fungal classes revealed a statistically significant tillage × season interaction term after correction for multiple testing (**Figure 3B**). Nevertheless, the majority of the bacterial phyla were not influenced by the tillage regime at the last growing stage investigated (grain filling for winter wheat). These results are in line with those shown in the **Table 2**.

**FIGURE 4:**
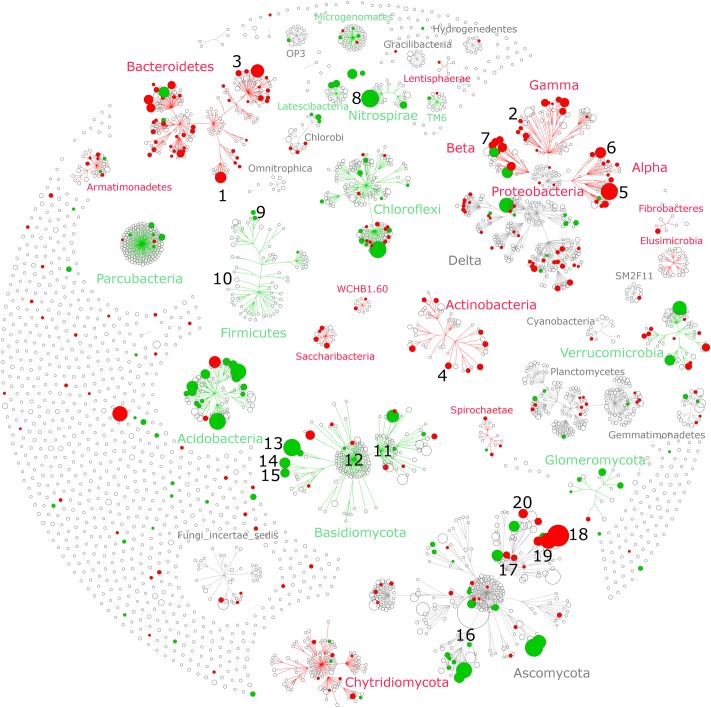
Taxonomic networks showing the distribution of bacterial and fungal OTUs across the taxonomic hierarchy. Nodes correspond to OTUs and node size corresponds to their relative abundance (square root) in the dataset. Edges (lines connecting the nodes) represent the taxonomic path from phylum to OTU level, whereas OTUs are placed at the level of the lowest possible assignment. The response of individual OTUs to tillage was mapped onto the taxonomic network with green nodes corresponding to OTUs responding positively to reduced tillage (RT) and red nodes corresponding to OTUs responding positively to conventional tillage (CT). Only significant nodes were color-coded (*q* < 0.05). The numbers (1–20) correspond to the most salient microbial groups showing a response to tillage and these are discussed in the section “Potential Interactions between Microbial Taxa and Their Environment” of the main text.

The individual relative change in abundance to tillage regime was also determined at the OTU-level. A total of 257 (15%) bacterial and 126 (8%) fungal OTUs showed a significant change in their relative abundance between CT and RT. Among those, 182 bacterial and 72 fungal OTUs responded positively to CT, whereas 75 bacterial and 54 fungal OTUs responded positively to RT. The distribution of these OTUs across the taxonomic hierarchy is shown in **Figure 4**. Several higher-order taxonomic groups such as Acidobacteria, Actinobacteria, Bacteroidetes, α- and β-Proteobacteria, and Glomeromycota showed a largely uniform response, i.e., most OTUs responding in the same direction to tillage, with a few exceptions. Other groups such as Verrucomicrobia, Chloroflexi, or Ascomycota showed a more heterogeneous response at the OTU level. On the basis of the existing scientific literature, the ecological relevance and potential lifestyles of the most salient tillage-sensitive taxa will be discussed in the next section; however, the statistics for all higher-order groups as well as OTUs including the unadjusted (p) levels of significance are provided in Supplementary Data Sheets S1, S2, respectively.

## Discussion

### The Effect of Tillage on Soil Microbial α- and β-Diversity

Here, we explored tillage-induced changes in soil microbial community structure below the seedbed under conventional and reduced tillage. At this depth (15–20 cm), the difference in soil physical and chemical conditions between CT and RT was expected to be higher than in the top soil, since the soils have been undisturbed for the last 6 years under RT. In this context, it is important to note that the initial impact and temporal dynamics of these effects could be different at other soils depths as shown before (Degrune et al., 2016). Overall, our findings evidenced that soils under CT host a more rich and less even bacterial and fungal community than under RT for both bacteria and fungi, whereas no interaction between tillage and growing season has been observed (**Figure 1B** and **Table 3**). Based on the *intermediate disturbance hypothesis* (IDH) or “hump-back model” that describe the response of a community to stress (Giller et al., 1998), it could be assumed that under CT, plowing may act as an intermediate disturbance that is neither too rare nor too frequent, and results in an increased OTU diversity. Plowing mixes the different horizons and breaks down soil aggregates, which in turn releases organic matter and creates new ecological niches that allow colonization through minor or new species (Tilman, 1982). Indeed, the disturbance under CT might enhance r-strategic microorganisms, resulting in a more uneven community dominated by OTUs that thrive more efficiently under increased availability of easily accessible nutrients. Our estimation of diversity, however, was based on specific periods of the growing season, which was from March to June. Therefore, our results cannot be extrapolated to conclude that the community under CT is consistently more rich and less even over the whole year. To answer this question, further analyses are needed where the estimation of diversity is based on the entire year. However, it is difficult to interpret shifts in richness and evenness with respect to ecosystem functioning and crop productivity as relatively rare species can strongly influence certain soil processes. Consequently, we focus our discussion on the change in β-diversity and the taxonomic identity of tillage-sensitive taxa as they can play a beneficial or detrimental role in agroecosystems (Aislabie et al., 2013).

In agreement with the first hypothesis of our study, the tillage regime was a significant driver of microbial β-diversity (**Figure 1A** and **Tables 2**, **3**), which is consistent with the recent literature using high-resolution techniques (Quadros et al., 2012; Navarro-Noya et al., 2013; Carbonetto et al., 2014; Sengupta and Dick, 2015; Degrune et al., 2016; Jiménez-Bueno et al., 2016). However, the direction of change of some microbial groups was not consistent with the other studies. For example, Carbonetto et al. (2014) reported higher relative abundance of Actinobacteria in no-tilled soil, whereas we evidenced higher abundance under CT when compared to RT (**Figure 3A**). In the same study, the relative abundance of Nitrospirae was higher in tilled soil, whereas in our study the Nitrospirae were higher under RT. Therefore, whereas there is a consensus that tillage alters soil microbial community structure, the response of individual groups appears to be very context-specific and cannot be generalized across various agroecosystems. The response is largely dependent on the soil physical and chemical conditions induced by the tillage regime, which again differs among different soil types and under different climatic conditions. It is also important to note that the sampling depth is often different among the studies and can lead to different results and conclusions.

In our field study, 6 years after conversion from conventional to reduced tillage, soil nutrient and moisture contents below the seedbed were significantly lower under RT (**Figure 2A**). Furthermore, previous investigations in the same field and at the same depth found that the soil’s resistance to penetration as an estimation of soil density was more than twice as high under RT (40 ± 6 kg cm^-2^) than under CT (15 ± 1 kg cm^-2^).

### The Effect of Tillage over the Growing Season

According to the second hypothesis, we expected that tillage effects vary across the growing season and that differences get smaller towards the end of the season. Indeed, the establishment of the root system over the season was expected to “dilute” the tillage regime effect on microbial β-diversity. For both bacteria and fungi, a moderate effect of this interaction was noticed when compared to the tillage regime effect (**Table 2A**). Moreover, for bacteria, the pairwise test revealed no tillage effect at the last stage of wheat. These results suggest that bacteria and fungi differ in their response to tillage over the growing season. Fungi showed less resilience and a stronger crop effect, whereas bacteria appeared to be more resilient over the course of the season. The moderate interaction effect was further evidenced by looking at the individual responses of higher-order taxonomic groups, where a certain response variability was observed across the growing season, but no statistically significant interactions were identified (**Figure 3**). It could be argued, however, that corrections for multiple testing were potentially too conservative.

An interaction between tillage and growing season would be expected as the establishment of the rooting system over time significantly influences the surrounding soil and may lead to changes in the carbon source (root exudation), pH (ions release or uptake), water and oxygen contents (root water uptake and respiration), and nutrient availability (plant uptake and secretion of chelators to sequester micronutrients) (Philippot et al., 2013). In addition, the soil structure that determines pore connectivity and associated fluxes of oxygen and water, is also influenced by the root system (Bronick and Lal, 2005). Other factors may also contribute to the interaction effect between tillage and growing season and include microbial resilience, i.e., the microbial community naturally recovers from the mechanical disturbance over time, as well as the climatic conditions that change over the season (e.g., temperature, moisture). The fact that only a moderate interaction effect was noticed might be linked to the sampling design. The samples were collected as close as possible to the stem, therefore the soil was not totally bulk soil, neither totally rhizosphere. We might assume that larger interaction effects would be detected, if samples were collected within the rhizosphere, i.e., the narrow region of soil that is directly influenced by root secretions.

Our findings further evidenced substantial variability in physical and chemical soil parameters over the growing season of the two crops between CT and RT below the seedbed (**Figure 2B**). The magnitude of tillage effect varied over time and differed across the studied parameters. This variability in the magnitude of changes between CT and RT over the growing season might be attributed to the establishment of the root system that differs between CT and RT and that in turn might influence the water and nutrient flows through the soil profile. Previous measurement on the same experiment identified that under RT, the rooting system was limited in the top soil mostly because of the presence of a highly compacted soil layer below 10 cm, whereas under CT the rooting system was less limited and explored the whole soil profile (Eylenbosch et al., 2015). Consequently, over the growing season, the soil under CT at the studied depth was colonized by roots, whereas the soil under RT was not. In addition, the flows of water and nutrients were likely to be altered under RT due to the compaction, thus leading to different penetration dynamics under CT and RT over the growing season.

The impact of tillage over the growing season on the structure of microbial communities was previously investigated using lower resolution methods such as biochemical assays (Spedding et al., 2004; Zhang et al., 2012; Shi et al., 2013). Interactive effects between tillage and growing season, however, were not consistent across these studies. Whereas Zhang et al. (2012) and Shi et al. (2013) reported that the tillage effect on the soil microbiota was dependent on the stage of growing season, Spedding et al. (2004) found no interaction effects between tillage and growing season. Again, the regional climatic conditions as well as local edaphic properties including soil texture, structure, and moisture, may explain the discrepancy across different studies. For example, soil texture is one of the major determinants of how soil (and its inhabitants) responds to mechanical disturbance (Hartmann et al., 2014) and the water regime driven by climate were also found to strongly influence microbial diversity (Drenovsky et al., 2004; Ulrich and Becker, 2006). Therefore, it remains difficult to draw universally valid conclusions in that respect.

### Potential Interactions between Microbial Taxa and Their Environment

The tillage regime caused substantial changes in soil physical (moisture and aeration), chemical (nutrient availability and carbon accessibility) and biological (root system development) conditions below the seedbed. In our study, substantial differences in nutrient and moisture contents were recorded below the seedbed, with CT featuring higher nutrient and moisture contents than RT (**Figure 2A**). As mentioned earlier, the absence of plowing for the last 6 years under reduced tillage has led to the formation of highly compacted soil layer, resulting in alteration of the soil pore network that in turn might influence the nutrient and water flows through the soil profile as well as root penetration into the lower soil layers. Consequently, we expect that nutrients and moisture largely remained in the first centimeters of soil under RT, while under CT, the penetration of nutrients and moisture in deeper layer was facilitated by the higher occurrence of macrospores resulting from the alteration of the soil pore network by plowing (Lipiec et al., 2006). The quantity of crop residues was also likely to be different between CT and RT, resulting in higher availability of C source under CT when compared to RT where crop residues remained at top soil. Moreover, the quality of C was also likely to be different between CT and RT, with more recalcitrant material under CT (fresh crop residues added yearly by plowing).

All these changes in physical and chemical parameters between CT and RT were expected to induce substantial changes in the structure of microbial communities. In our first hypothesis, we speculate on the presence of some taxa in relation with soil physical and chemical conditions found under CT and RT. Here, we used the taxonomic information to infer on the presence of some taxa displaying specific lifestyles that can be related to environmental characteristics. Although such information on lifestyles can be found at higher taxonomic levels (Philippot et al., 2010), there is an interest to go deeper in the taxonomy and identify members involved in more complex functions usually shown at lower taxonomic levels (Martiny et al., 2013). To date, however, describing the entire diversity of microbial communities with respect to the changes in environmental factors remains a challenge since we still have a limited understanding of the ecological attributes of many microbial taxa and many OTUs cannot be assigned at lower taxonomic levels. Consequently, we focused our analysis on the most salient examples.

According to the oligotrophy-copiotrophy framework previously outlined by Fierer et al. (2007), the higher nutrient status found under CT might explain the higher relative abundance of Proteobacteria (α, β, and γ) and Bacteroidetes, bacterial groups that reportedly feature mainly copiotrophic lifestyles and thrive better under conditions of high nutrient availability. In contrast, the lower nutrient status found under RT might explain the higher relative abundance of Acidobacteria, which was reported to largely exhibit oligotrophic lifestyles and thrive better under conditions of lower nutrient availability (Fierer et al., 2007, 2012; Rasche et al., 2011).

Several bacterial groups that are known to carry the ability to degrade recalcitrant C compounds such lignin found in crop residues, including α-, γ-, and β-Proteobacteria, as well as Actinobacteria (Goldfarb et al., 2011; Kameshwar and Qin, 2016), were significantly increased under CT. The putative ability to degrade complex organic matter under CT was also found at lower taxonomic resolution (**Figure 4**). Some members of genera *Flavobacterium* (**Figure 4**, clade #1) and *Cellvibrio* (clade #2), are known to be involved in lignocellulose degradation (Koga et al., 1999; Jiménez et al., 2013; Burgess, 2015), members of genus *Adhaeribacter* (clade #3) showed increase in soils receiving organic amendments, suggesting efficient usage of complex organic matter (Calleja-Cervantes et al., 2015), and members of *Actinoplanes* (clade #4) are found to be more abundant in leaf litter samples (Binh et al., 2011).

Bacterial groups from α- and β-Proteobacteria that are important drivers of crop productivity were significantly increased under CT. The order Rhizobiales (clade #5) as well as the genus *Sphingomonas* (clade #6) and *Rhizobacter* (clade #7) are a well-known plant growth promoting rhizobacteria (PGPR) that stimulate plant growth through various mechanisms such as biocontrol and nitrogen fixation (Haas and Défago, 2005; Tsavkelova et al., 2006; Zandi and Basu, 2016). In contrast to our results, recent studies evidenced a positive effect of conservation tillage (zero-tillage) on the relative abundance of Rhizobiales (Ceja-Navarro et al., 2010; Souza et al., 2013). Our results emphasized again the importance of the local pedologic and climatic context that drive the effect of tillage regime on soil physical and chemical conditions that in turn influence the abundance of beneficial microorganisms.

In RT soils, we identified an increased abundance of the phylum Nitrospirae (**Figure 3A**) and its genus Nitrospira (**Figure 4**, clade #8). Members of this group are nitrite-oxidizing bacteria (Juretschko et al., 1998; Daims et al., 2001) and exhibit largely oligotrophic characteristics (Schramm et al., 1999; Nowka et al., 2015). In addition, a recent study identified an increase of Nitrospirae in compacted soils (Hartmann et al., 2014) such that we can speculate that the increase relative abundance of Nitrospirae under RT indicated that these soils are less aerated than under CT as suggested by the strong difference in soil density mentioned above. At phylum level, Firmicutes were found to be more abundant under RT, but at OTU level, only a few responded to tillage regime. Among them, we identified three OTUs responding positively to RT that were associated with endospore forming taxa such as *Paenibacillus* (aerobic) (clade #9) and *Clostridium* (anaerobic) (clade #10). Members of the Clostridiales (containing *Clostridium*) are metabolically diverse and may ferment sugars, starch, pectin, and cellulose under more oxygen-limited conditions (Goldfarb et al., 2011). Here again, we can speculate that an increased relative abundance of *Clostridium* could indicate more anaerobic microsites under RT.

Several groups of the recently suggested candidate phyla radiation (CPR) (Brown et al., 2015) differed in abundance between the tillage regimes. In general, members of the CPR have small streamlined genome, are versatile in their nutrient-spectrum (Wrighton et al., 2012), and exhibit potentially ectosymbiotic lifestyles, i.e., living on the surface of the host (Nelson and Stegen, 2015; Yeoh et al., 2015). These characteristics appear to lead to adaptation to more nutrient poor, oligotrophic conditions, as they have even been found to be strongly enriched in highly oligotrophic environments such as permafrost (Frey et al., 2016) and deep sea sediments (Zhu et al., 2013). Therefore, we can speculate that the increased relative abundance of Parcubacteria (formerly OD1) and Microgenomates (formerly OP11) under RT (**Figure 4**) is another indication that these soils are likely more nutrient-limited than under CT.

Fungi, known as major drivers of organic matter decomposition, showed substantial variability in community structure between CT and RT and they also showed less resilience towards the end of the growing season (**Table 2B**). Although fungi are often sensitive to mechanical disturbance that cause damages to their hyphal network, some major groups such as Chytridiomycota and Sordariomycetes (major class of Ascomycota) depicted higher abundance under CT (**Figure 3A**). Since many fungi use crop residues as a substrate, one would expect to see differences in fungal community structure caused by the different qualities of crop residues in deeper soil between CT and RT. However, our results showed no overall effect of crop residues on fungal diversity.

Members of Chytridiomycota are commonly found in soil and exhibit either saprobic or parasitic lifestyles, but the ecological relevance of Chytridiomycota in agroecosystems is still poorly understood. Most of them are unicellular and only few show multicellular hyphal growth, which could be one reason why they are relatively more abundant under CT as they might be less susceptible to mechanical disturbance. A recent study have emphasized their potential ability to degrade cellulose, a major component of plant cell wall, suggesting an important role in C-decomposition (Kameshwar and Qin, 2016).

Basidiomycota, a vast and complex group of fungi containing a large number of saprophytic (wood decayers, litter decomposer), ectomycorrhizal, and parasitic fungi (Watkinson, 2008), was found to be higher under RT (**Figure 3A**). Most of the abundant members of this phylum responded positively to reduced tillage (**Figure 4**). Typically, saprophytic members were recognized to degrade complex components such as lignin contained in plant litter and wood more rapidly than other fungi (Osono and Takeda, 2002). The two major classes of Basidiomycota belong to Agaricomycetes (**Figure 4**, clade #11) and Tremellomycetes (**Figure 4**, clade #12), and responded positively to RT (**Figure 3A**). Notably, Agaricomycetes are critical decomposers and contain the ‘soft,’ ‘brown,’ and ‘white’ rot fungi that produce hydrogen peroxide and enzymes to degrade complex plant compounds including cellulose and lignin (Kameshwar and Qin, 2016). At finer taxonomic resolution we identified three major fungi including *Guehomyces pullulans* (clade #13), and two species of *Cryptococcus* (*C. terricola* and *C. aerius*) (clades #14 and #15). These organisms are single-celled microorganisms (yeast) and known to feature a wide range of enzymatic activities (Martinez et al., 2016). Yeast have developed adaptation strategies to overcome notably low-nutrient and oxygen-poor conditions (Fonseca and Inácio, 2006), for instance those found in oligotrophic lake in Patagonia (Brandão et al., 2011) and glacial areas (Buzzini et al., 2012). Again, the presence of such oligotrophic organisms might be related to the more nutrient- and oxygen-limited conditions found under RT when compared to CT.

The Glomeromycota, a fungal group of significant ecological and economic importance, was found to be more abundant under RT. Members of this group contain arbuscular mycorrhizal fungi (or AMF) that form symbiotic associations with the majority of vascular plants and significantly increase nutrient availability for the host plant, and, thus, play a crucial role in agroecosystem functioning (Douds and Millner, 1999). It has previously been shown that this group of fungi is enhanced under reduced tillage (Säle et al., 2015).

Ascomycota display a large and wide range of lifestyles. Although no overall tillage effect on this group was noticed (**Figure 3A**), the individual OTUs belonging to Pezizales responded uniformly and positively to RT (**Figure 4**, clade #16), whereas the response of individual OTUs within Sordariomycetes was less uniform (clade #17). Sordariomycetes (clade #17) is one of the largest class of Ascomycota and feature a wide range of lifestyles such as pathogens and endophytes of plants, and mycoparasites (Zhang et al., 2006). Although this group responded positively to CT (**Figure 3A**), the individual response to tillage at the OTU level differed substantially (**Figure 4**, clade #17). Among the most abundant OTUs, members of the genera *Podospora* and *Schizothecium* were identified to be more abundant under CT (**Figure 4**, clade #18 and #19). Both of them, phylogenetically similar (Cai et al., 2005), belong to coprophilous, a type of saprobic fungi that grow on animal dung. We further identified *Fusarium graminearum* (**Figure 4**, clade #20), the causative agent of Fusarium head blight of wheat (Bottalico and Perrone, 2002), to be more abundant under CT. As reported by Booth (1971), *F. graminearum* can survive saprophytically on a wide range of gramineous host debris, such as wheat residues. As our samples were taken at a depth between 15 and 20 cm, the higher relative abundance of *F. graminearum* observed under CT might be due to the presence of crop residues from previous wheat crops at this depth, while crop residues remain in the topsoil (<10 cm) under RT.

## Conclusion

In our study, we have evidenced a substantial effect of the tillage regime on soil microbial community structure below the seedbed. We have also shown a moderate dependency of these tillage effects on the growing season. Changes in microbial community structure were related to variation in soil physical and chemical properties, with soil under reduced tillage being more nutrient-, water-, and oxygen-limited than under conventional tillage. In the local climatic and pedologic context found in cropping systems of central Belgium, implementing reduced tillage might have led to detrimental effects on soil quality. More specifically, soil compaction occurring in the upper soil layer has restricted the establishment of the root system with possible negative consequences for crop productivity. The altered physicochemical conditions under the different tillage regimes have promoted microorganisms with different lifestyles. Reduced tillage appeared to promote organisms that thrive under more limited conditions, but we currently have no evidence, if this shift actually affects soil functioning and influences crop productivity and crop health. In order to increase our understanding of the relationships between changes in microbial community structure and plant productivity and health, a functional approach should be used to complement the knowledge gained by the taxonomic survey.

## Author Contributions

Conceived and designed the experiments: GC, BB, and MV. Management of the experiment: M-PH. Soil sampling: FD. DNA-amplicon sequencing: BT and GD. Analyzed the data: FD and MH. Wrote the papers: FD and MH. All authors reviewed the manuscript.

## Conflict of Interest Statement

The authors declare that the research was conducted in the absence of any commercial or financial relationships that could be construed as a potential conflict of interest.
